# Regulation of APC/C-Cdh1 and Its Function in Neuronal Survival

**DOI:** 10.1007/s12035-012-8309-2

**Published:** 2012-07-27

**Authors:** Angeles Almeida

**Affiliations:** 1 Instituto de Investigación Biomédica de Salamanca, Hospital Universitario de Salamanca, 37007 Salamanca, Spain; 2 Departamento de Bioquímica y Biología Molecular, Instituto de Neurociencias de Castilla y León, Universidad de Salamanca, 37007 Salamanca, Spain; 3 Research Unit, University Hospital of Salamanca, Edificio Departamental (Lab. 122), Plaza Doctores de la Reina, 37007 Salamanca, Spain

**Keywords:** APC/C, Cdh1, Cell cycle regulation, Neuronal survival, Neurodegeneration

## Abstract

Neurons are post-mitotic cells that undergo an active downregulation of cell cycle-related proteins to survive. The activity of the anaphase-promoting complex/cyclosome (APC/C), an E3 ubiquitin ligase that regulates cell cycle progression in proliferating cells, plays a relevant role in post-mitotic neurons. Recent advances in the study of the regulation of APC/C have documented that the APC/C-activating cofactor, Cdh1, is essential for the function(s) of APC/C in neuronal survival. Here, we review the normal regulation of APC/C activity in proliferating cells and neurons. We conclude that in neurons the APC/C-Cdh1 complex actively downregulates the stability of the cell cycle protein cyclin B1 and the glycolytic enzyme 6-phosphofructo-2-kinase/fructose-2,6-bisphosphatase-3. Keeping these proteins destabilized is critical both for preventing the aberrant reentry of post-mitotic neurons into the cell cycle and for maintaining their reduced antioxidant status. Further understanding of the pathophysiological regulation of these proteins by APC/C-Cdh1 in neurons will be important for the search for novel therapeutic targets against neurodegeneration.

## Introduction

The cell cycle is an essential process for the development, differentiation, and proliferation of eukaryotic cells. Classically, the cell cycle is divided into four phases, namely, G1, S, G2, and M. Proper progression from one phase to the other is monitored by checkpoints that sense possible defects during DNA synthesis and chromosome segregation [1]. Progression through the cell cycle is controlled by the appropriate and timely activation of cell cycle proteins. This control is executed by the ubiquitin proteasome system (UPS). The UPS consists of sets of enzymes that target substrates to proteasomal destruction by covalently attaching a polyubiquitin chain in an enzymatic cascade [2]. First, ubiquitin is covalently linked to the E1 ubiquitin-activating enzyme in an energy (ATP)-dependent manner. Ubiquitin is then transferred to the E2 ubiquitin-conjugating enzyme. Finally, the E3 ubiquitin ligase recruits both the E2 ubiquitin-conjugating enzyme and substrate, thus facilitating the transfer of ubiquitin to the lysine residues of the substrate. Polyubiquitinated substrates are then recognized by the proteasome, which leads them to degradation [2].

The specificity of the UPS is conferred by a large and varied collection of E3 ubiquitin ligases. The cullin-really interesting new gene (RING)-finger-type E3s constitute one of the largest classes of E3s and are essential for the maintenance of genomic integrity and cellular homeostasis [3]. Two major cullin-RING-E3 ubiquitin ligases, the SKP1-CUL1-F-box-protein (SCF) complex and the anaphase-promoting complex, also called the cyclosome (APC/C), are responsible for targeting cell cycle proteins for degradation. Whereas SCF ubiquitylates substrates from late G1 to early M phase, the APC/C is active from the onset of mitosis to the end of the G1 phase of the cell cycle [4, 5]. Differentiated cells, such as neurons, remain resting in the G0 phase due to an active downregulation of cell cycle-related proteins. However, recent evidence has indicated that neurons retain the ability to reactivate the cell cycle in response to central nervous system (CNS) insults. Thus, neurons attempt to reenter the cell cycle under pathological circumstances, including both acute injury and chronic neurodegenerative disorders [6–8]. For instance, in vivo evidence has revealed S phase entry in ischemic neurons [9] and aberrant expression of mitotic cyclin B1 in degenerating neurons in Alzheimer’s disease [10]. Thus, dysregulation of the cell cycle machinery might be a pathway common to several neurodegenerative disorders and other CNS diseases [11]. It has recently been reported that APC/C activity, which regulates cell cycle progression in proliferating cells [12–14], is also essential for neuronal survival [15, 16], linking proliferation to neurodegeneration. This review summarizes recent findings describing the functions of APC/C in neuronal cell death.

## The APC/C Complex

The APC/C is a multi-subunit cullin-RING E3 ubiquitin ligase assembled from 13 different core subunits that regulates progression from metaphase to anaphase and exit from mitosis [12–14, 17]. Among these subunits, the best-characterized ones are the cullin and RING proteins Apc2 and Apc11, which are responsible for catalytic activity, the tetratricopeptide (TPR) repeat subunit Apc3 (or Cdc27), which interacts with co-activators, and the Apc10, also known as Doc1 [17, 18]. The RING-finger protein Apc11 interacts directly with the Ub-conjugated E2 enzyme, while the cullin domain of Apc2 interacts with the RING-finger domain of Apc11, acting as a scaffold to connect Apc11 to the enzyme [19].

## Regulation of the APC/C in the Cell Cycle

The activity of the APC/C is tightly regulated along the cell cycle. This is exerted through a combination of co-activator subunits, reversible phosphorylation, and inhibitory proteins and complexes [12–14]. To be active, the APC/C requires the binding of either one of two WD40-domain co-activator proteins, Cdc20 or Cdh1, which also participate in substrate recognition [20–22]. The activators interact dynamically with the TPR domains in Apc3 and may either facilitate recruitment of substrates through their WD40 domains or enhance the specific activity of the APC/C [23]. The TPR subunit Apc3 also interacts with the Apc10 subunit [24]. Apc10 contains a β-barrel structure, known as the Doc domain that is involved in substrate binding and recognition [25, 26].

Activation of the APC/C by Cdc20 or Cdh1 is regulated tightly and oppositely by phosphorylation events. In early mitosis, cyclin–cyclin-dependent kinase (Cdk) complexes phosphorylate some APC/C subunits, hence promoting the binding of Cdc20 to the complex [27]. APC/C-Cdc20 thus initiates a sequence of degradation of mitotic cyclins that results in decreased Cdk activity, leading to the initiation of anaphase. In contrast, the phosphorylation of Cdh1 by Cdks during S phase, G2, and mitosis inhibits its binding to the APC/C [28, 29]. During exit from mitosis, the inactivation of Cdks and subsequent activation of phosphatases [30] allows Cdh1 dephosphorylation, leading to the activation of the APC/C-Cdh1 complex. In turn, APC/C-Cdh1 ubiquitylates Cdc20, thus preventing the simultaneous activation of both APC/C co-activators. APC/C-Cdh1 also targets A/B types of mitotic cyclins for destruction, completing the inactivation of Cdk1. In particular, cyclin B1 is degraded in two phases, sequentially regulated by APC/C-Cdc20 and APC/C-Cdh1 [31, 32]. Subsequently, during early mitosis, Cdc20 activates APC/C, whereas in late mitosis APC/C-Cdh1 activation controls mitotic exit and G1 maintenance, thus regulating the onset of DNA replication [12–14].

## Substrate Recognition by APC/C-Cdh1

APC/C-Cdc20 and APC/C-Cdh1 have different substrate specificities, which allow the orchestration of cell cycle protein degradation in the correct order. Selection of the APC/C targets is controlled by the recognition of short degron motifs, predominantly the destruction (D) box (RxxLxxxxN) and the KEN box (KENxxxN) [17]. The D box was first described to be necessary and sufficient for the APC/C-mediated ubiquitylation of B type cyclins [33–35]. The KEN motif is commonly present in APC/C substrates, usually in addition to the D box [36]; however, some substrates contain only either a D box or KEN box, in one or more copies each. While the D box is recognized by both APC/C-Cdc20 and APC/C-Cdh1, the latter also recognizes targets with a KEN box motif [33, 36, 37]. Although the presence of D and KEN box sequences in proteins is required for APC/C-mediated ubiquitination of many substrates, it is often not sufficient. Other less known motifs, such as the A box (RxLxPSN), the CRY (CRYxPS) box, and the KEN-like GxEN box, have also been identified [38]. For instance, Aurora A contains both a D box and a KEN box, but its recognition by APC/C-Cdh1 also requires the destruction motif A box [39]. Furthermore, the APC/C-Cdh1-dependent destruction of Cdc20 requires not only its KEN box but also its CRY box [40]. Finally, Castro et al. [41] identified a GxEN box within the C-terminus of XKid, a protein involved in chromosome segregation in anaphase; this GxEN box being necessary for its recognition and association with APC/C.

Yamano et al. [42] described a direct interaction of the APC/C core with substrates via the degron motifs. The core subunit Doc1/Apc10 appears to regulate the direct interaction and binding of specific substrates [43, 44]. Site-directed mutagenesis analyses in Apc10/Doc1 demonstrated that this subunit specifically mediates D box-dependent recognition [25]; however, Apc10/Doc1 does not participate in the recognition of KEN box by Cdh1. Recently, da Fonseca et al. [26] have reported that Cdh1 and Doc1/Apc10 function as the D box co-receptor.

## Phosphorylation of Cdh1 and APC/C Activity

**Fig. 1:**
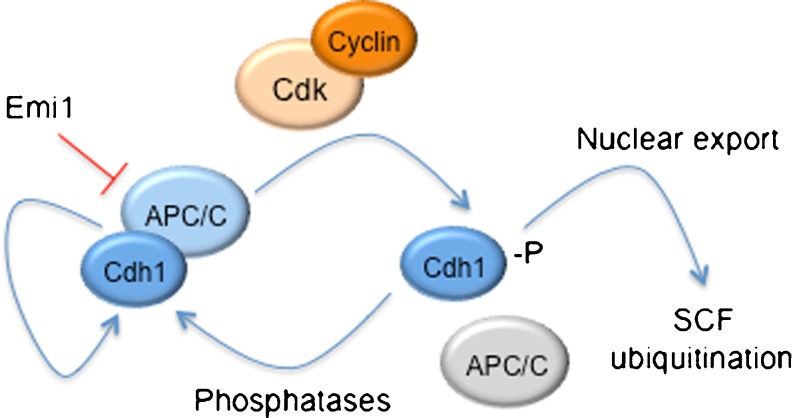
Regulation of APC/C-Cdh1 activity. The activity of APC/C-Cdh1 is tightly regulated by reversible phosphorylation of Cdh1, inhibitor proteins, and Cdh1 degradation. The phosphorylation of Cdh1 by cyclin-Cdks blocks its own interaction and binding to APC/C. Cdh1 dephosphorylation triggers APC/C-Cdh1 activation. Phosphorylated Cdh1 is translocated from the nuclei to the cytosol where it is recognized and targeted for degradation by the SCF complex. Cdh1 is also inhibited by Emi1, which acts as a pseudosubstrate that inactivates APC/C-Cdh1 at G2 and G1/S transition. Finally, Cdh1 mediates its own degradation by stimulating APC/C activity, which depends upon two D boxes

The activity of APC/C-Cdh1 increases in late anaphase and is maintained elevated through the G1 phase of the cell cycle, and it is tightly regulated through the reversible phosphorylation of Cdh1 and its substrates, inhibitor proteins, and Cdh1 degradation (Fig. 1). The phosphorylation of Cdh1 by Cdk1 and Cdk2, which starts from G1/S transition to anaphase, blocks its own interaction and binding to APC/C [28]. Recently, it has been described that Cdk5, a member of the CNS predominant Cdk family that is essential for synaptic plasticity [45] and neurotoxicity [46, 47], phosphorylates (and inactivates) Cdh1 [16].

It is known that Cdh1 dephosphorylation depends not only on Cdk inactivation but also on phosphatase activation [30]. In budding yeast, the main Cdh1 phosphatase, Cdc14, mediates the activation of both Cdh1 and the Cdk1 inhibitor protein Sic1, leading to the completion of Cdk1 inactivation [48, 49]. However, although three homologues of yeast Cdc14 (CDC14A, CDC14B, and CDC14C) have been identified in vertebrates, their relevance remains unclear, given the lack of a clear effect on mitotic exit [50–53]. By contrast, mitotic exit in animal cells is independent of Cdc14 and, instead, relies on phosphatases of the PP1 and PP2A families [30]. In this context, numerous studies suggest functions of vertebrate Cdc14 that are unrelated to mitotic exit, including roles in the DNA damage checkpoint [54], DNA repair [53], and centrosome duplication and function [51, 55]. In particular, in response to genotoxic stress in G2, Cdc14B translocates from the nucleolus to the nucleoplasm, where this phosphatase dephosphorylates Cdh1, hence promoting APC/C-Cdh1 activity [54].

The phosphorylation of Cdh1 substrates also contributes to the regulation of APC/C-Cdh1 activity. For example, Cdk2-mediated phosphorylation of the SCF E3 ubiquitin ligase cofactor, Skp2, disrupts its association with APC/C-Cdh1, leading to Skp2 accumulation at the end of G1 phase [56, 57]. On the other hand, Cdc14B-dependent dephosphorylation of Cdh1 promotes Skp2 degradation at the M/G1 transition [58].

In addition to inhibitory phosphorylation by Cdks, Cdh1 is also inhibited by Emi1, which acts as a pseudosubstrate that inactivates APC/C-Cdh1 at G2 and the G1/S transition [59–61]. Emi1 contains a D box motif that mediates its own binding to APC/C-Cdh1, thus blocking the accessibility of substrates to the enzyme. Moreover, Emi1 also contains a ZBR motif that directly inhibits APC/C E3 ligase activity [62].

The subcellular localization of Cdh1 provides an important element of spatial regulation of APC/C-Cdh1 activity [63, 64]. Cdh1 is located in the *nuclei* during G1 but redistributes to the cytosol between S phase and the end of mitosis [63]. Nuclear export of Cdh1 to the cytosol is promoted by Cdk phosphorylation [16, 63], which sequesters Cdh1 from either the APC/C or nuclear targets, thus contributing to the efficient inactivation of APC/C-Cdh1. Furthermore, phosphorylated Cdh1 is recognized and targeted for degradation by the SCF complex, the predominant E3 ubiquitin ligase in S phase [65].

Finally, Listovsky et al. [66] have described that APC/C activity is regulated by Cdh1 protein abundance. The levels of Cdh1 are high in mitosis, when it is inactive; however, when Cdh1 is active in G1 and G0, its levels are considerably lower, although mostly associated with APC/C. Those authors concluded that Cdh1 mediates its own degradation by stimulating APC/C activity, which depends upon two D boxes [66]. In addition, Rape and Kirschner [67] reported that APC/C-Cdh1-mediated proteasomal degradation of the ubiquitin-conjugating enzyme (E2) UbcH10 also provides a negative feedback mechanism that inactivates APC/C-Cdh1 during the G1/S transition.

## APC/C-Cdh1 in G0/G1 Regulation

As mentioned above, cyclin-Cdk complexes phosphorylate Cdh1, inhibiting its binding to the APC/C complex [28, 29]; the concomitant inhibition of APC/C activity is released when cyclin-Cdk activity decreases and Cdh1 phosphorylation stops in late mitosis. This leads to APC/C-Cdh1 reactivation, which in turn maintains the cyclin B-Cdk1 inactive during late mitosis [68]. In this phase, APC/C-Cdh1 also mediates the degradation of regulators of cytokinesis and centrosome replication, such as Aurora A, Aurora B, Plk1, Anillin, and Tpx2, among other proteins [69, 70].

During G1, APC/C-Cdh1 remains active by maintaining the activity of cyclin-Cdk complexes low. In this context, active APC/C-Cdh1 targets A/B types of mitotic cyclins for proteasomal degradation [71, 72]. Moreover, APC/C-Cdh1 can also inactivate Cdk activity by promoting the degradation of the Cdk activator, Cdc25A, and the two cofactors of the SCF E3 ubiquitin ligase: Skp2 and Cks1. This leads to the accumulation of Cdk inhibitors p21, p27, and p57 [56, 57]. APC/C-Cdh1 also limits the expression of cyclin D1 through the elimination of the transcription factor Ets2 [73]. Thus, depletion of Cdh1 by siRNA has been shown to stabilize Skp2 and Ets2, resulting in p21 and p27 degradation and cyclin D1 elevation in G1, followed by a shorter G1 phase and premature entry into S phase [56, 73, 74]. Moreover, in Cdh1-deficient cells, the shortening of the G1 phase is associated with a prolonged and defective S phase [74–76]. As will be discussed below, in post-mitotic neurons, Cdh1 depletion causes a reentry into the S phase of the cell cycle followed by apoptotic cell death [15].

**Fig. 2:**
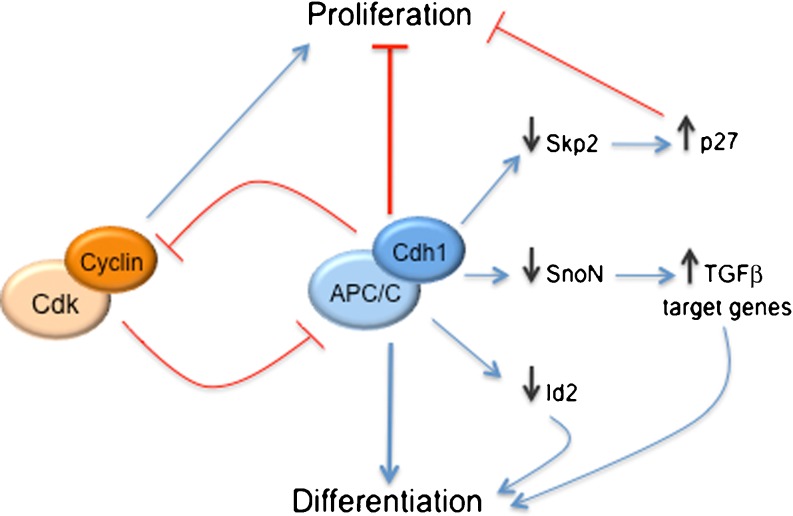
APC/C-Cdh1 coordinates cell cycle arrest and cellular differentiation. APC/C-Cdh1 inhibits cell proliferation by promoting the degradation of Cdk activators, such as cyclins, and the stabilization of Cdk inhibitors, including p27. APC/C-Cdh1 targets Skp2 for proteasomal degradation, leading to p27 accumulation and cell cycle arrest. Cyclin-Cdk complex phosphorylates Cdh1 and prevents APC/C activation. TGFβ stimulates APC/C-Cdh1-mediated SnoN degradation, leading to activation of TGFβ target genes and growth inhibition. APC/C-Cdh1 also triggers Id2 (inhibitor of differentiation/DNA binding 2) degradation. This promotes the activation of basic helix-loop-helix transcription factors and targets gene expression that mediates cellular differentiation

During G1, most cells commit alternatively to DNA replication and division or to cell cycle exit and differentiation. Because of its crucial role in G1/G0 regulation and maintenance of quiescence, APC/C-Cdh1 has been proposed to be responsible for linking cell cycle exit and differentiation in certain cell types [14, 38] (Fig. 2). Bar-On et al. [77] have recently described that APC/C-Cdh1-mediated Skp2 degradation, and the subsequent p27 accumulation are essential for human embryonic stem cell differentiation.

APC/C-Cdh1 is also crucial for the coordination of cell cycle progression and the initiation of lens [78] and muscle cell [79] differentiation. APC/C-Cdh1-regulated lens differentiation is mediated by transforming growth factor (TGF)β-induced destruction of SnoN, a transcriptional repressor of the Cdk inhibitors p15 and p21 (Fig. 2). Thus, depletion of Cdh1 by RNA interference attenuates the TGFβ-mediated induction of p15 and p21 and blocks lens differentiation [78]. Furthermore, TGFβ induces Skp2 degradation mediated by the Smad cascade, which stabilizes p27 and p21 and thereby contributes to TGFβ-induced cell cycle arrest [80]. In muscle, APC/C-Cdh1 regulates two critical proteins, Skp2 and Myf5, for proteolysis during muscle differentiation [79]. On the one hand, the targeting of Skp2 by APC/C-Cdh1 for destruction results in an accumulation of p21 and p27, which are crucial for coordinating cellular division and differentiation. On the other hand, the degradation of Myf5 facilitates myogenic fusion.

## APC/C-Cdh1 in Neuronal Differentiation

The activation of APC/C-Cdh1 is also required for neuronal differentiation. It has been reported that APC/C-Cdh1 mediates retinoic acid-induced neuronal differentiation from SH-SY5Y neuroblastoma cells [74]. Thus, retinoic acid induces the nuclear accumulation of Cdh1 that parallels Skp2 destabilization and p27 accumulation. Furthermore, retinoic acid decreases the mRNA and protein levels of Rae1—a nuclear export factor that limits APC/C-Cdh1 activity in mitosis—hence facilitating APC/C-Cdh1-mediated Skp2 degradation, leading to cell cycle arrest and neuroblastoma differentiation [74]. The regulation of this Skp2-p27 axis by APC/C-Cdh1 has also been found to be involved in the terminal differentiation of neuronal precursors in response to nerve growth factor [81].

Finally, APC/C-Cdh1 regulates axonal growth and patterning through the degradation of two nuclear proteins, namely, the inhibitor of differentiation/DNA binding 2 (Id2) and SnoN [82, 83] (Fig. 2). In the developing nervous system, Id2 enhances cell proliferation, promotes tumor progression, and inhibits the activity of neurogenic basic helix-loop-helix transcription factors [84]. Lasorella et al. [82] demonstrated that APC/C-Cdh1 targets Id2 for degradation through a D box motif that is conserved in Id1 and Id4. Degradation of Id2 in neurons permits the accumulation of the Nogo receptor and the subsequent inhibition of axonal growth. Furthermore, Stegmuller et al. [83] found that the Cdh1-dependent inhibition of TGFβ-SnoN axis stimulates axonal growth.

## Cyclin B1 Stability Regulation by APC/C-Cdh1 in Neuronal Survival

**Fig. 3:**
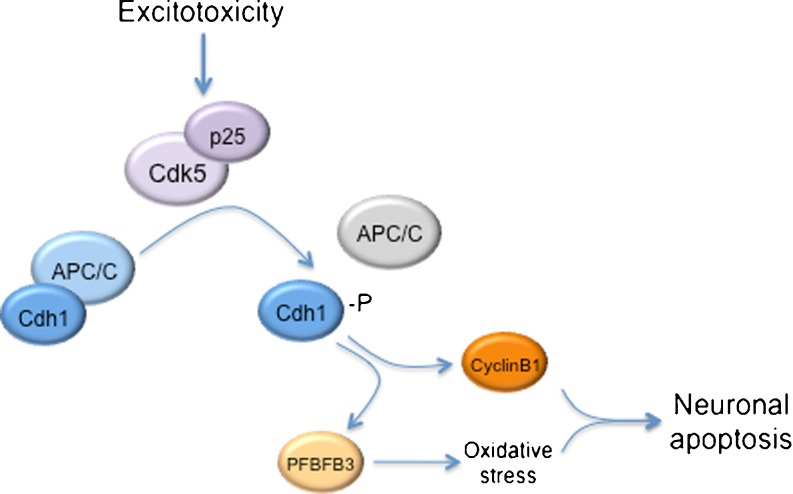
APC/C-Cdh1 regulates neuronal apoptosis. Excitotoxicity-induced Cdk5-p25 activation phosphorylates Cdh1. APC/C-Cdh1 inactivation causes the stabilization of the cell cycle protein cyclin B1 and the glycolytic enzyme 6-phosphofructo-2-kinase/fructose-2,6-bisphosphatase-3 (PFKFB3). Stabilization of these proteins induces both aberrant reentry of post-mitotic neurons into the cell cycle and oxidative stress, leading to neuronal apoptosis

Several core subunits of APC/C and Cdh1 are highly expressed in mammalian brain post-mitotic neurons [15, 85]. In neurons, depletion of Cdh1 by RNA interference rapidly triggers apoptotic neuronal death. Thus, APC/C-Cdh1 is required to maintain low levels of cyclin B1 in order to prevent an aberrant entry into the cell cycle that will lead to neuronal apoptosis (Fig. 3). Cdh1 then regulates neuronal survival [15]. Under physiological conditions, glutamate receptor-mediated excitatory neurotransmission plays a central role in neural development, differentiation, and synaptic plasticity. However, excessive or prolonged activation of glutamate receptors induces neurotoxicity, a process that has been defined as excitotoxicity [86, 87]. Excitotoxicity mediates neuronal death in several neurological disorders, including stroke and chronic neurodegenerative diseases [87, 88]. In a previous study, the changes in cyclin B1 protein levels following an excitotoxic stimulus caused by a short (5 min) incubation of post-mitotic cortical neurons with glutamate (100 μM) or the specific glutamate receptor subtype agonist, *N*-methyl-d-aspartate (NMDA; 100 μM) were investigated. Under these conditions, neurons died in an NMDA receptor-dependent manner [89]. Western blot analyses of neurons revealed that both glutamate and NMDA promoted an accumulation of cyclin B1 in the neuronal nuclei that was fully prevented by silencing cyclin B1 [16]. Furthermore, cyclin B1 nuclear accumulation was responsible for a large proportion of the apoptotic death caused by glutamate and NMDA. It was also found that glutamate and NMDA triggered Cdh1 phosphorylation, sequestering Cdh1 in the cytosol and inhibiting APC-Cdh1 activity.

Using both the Cdk inhibitor roscovitine and an RNA interference strategy, it was also demonstrated that Cdh1 was phosphorylated by Cdk5, an enzyme that can be persistently activated when bound to p25 [46], the proteolytic product of p35 that has previously been shown to accumulate in the neurons of patients with Alzheimer’s disease [90]. It was found that Cdk5 small interfering RNA dose-dependently prevented the apoptotic neuronal death triggered by NMDA and glutamate. These data were the first to suggest that NMDA receptor stimulation activates Cdk5, which phosphorylates Cdh1, leading to cyclin B1 accumulation and neuronal apoptotic death [16]. It is interesting to note that Cdk5 phosphorylates the NMDA receptor NR2A subunit at Ser-1232, hence facilitating NMDA receptor synaptic transmission [91]. Thus, NMDA receptor overactivation, which occurs in prolonged release of the neurotransmitter glutamate such as that occurring in neurodegenerative diseases and stroke [88], could lead to a Cdk5-NMDA receptor activation feedback loop, contributing to the propagation of neurodegeneration. Interestingly, cyclin B1 accumulates in degenerating brain areas in Alzheimer’s disease [10, 92] and stroke [93], which are situations known to be associated with an excitotoxic-type neuronal death [88]. Thus, these data suggest that Cdk5-mediated Cdh1 inactivation might contribute to neuronal death in neurological disorders.

## APC/C-Cdh1 in Glucose Metabolism and Oxidative Stress: Role in Neurodegeneration

Neurons are the highest energy-consuming cells of the brain; however, the rate of glycolysis, the metabolic route responsible for the generation of most energy needs in cells, is very low in neurons, particularly in comparison with their neighboring astrocytes [94, 95]. Glucose-derived glucose-6-phosphate is the connecting metabolite between glycolysis and the pentose-phosphate pathway (PPP) [96]. The PPP is the main metabolic pathway responsible for the regeneration of NADPH(H^+^), a required reducing cofactor for many oxidoreductases. Previously, it has been found that in cortical neurons the protein abundance of the glycolytic enzyme 6-phosphofructo-2-kinase/fructose-2,6-bisphosphatase, isoform 3 (PFKFB3) is very low in neurons as compared with that found in astrocytes [95]. This was considered important, since PFKFB3 is a key enzyme controlling the rat of glycolysis, and hence energy generation from glucose. Furthermore, it was found that overexpressing PFKFB3 in neurons increased the rate of glycolysis, although it concomitantly decreased that of the PPP [97].

Interestingly, PFKFB3 protein was found to be a substrate of APC/C-Cdh1 [97]. Accordingly, APC/C-Cdh1 activity determines the control of the rate of glucose consumption both through glycolysis and the PPP. In contrast to neurons, astrocytes express a very low Cdh1 protein abundance, and hence APC/C activity is negligible in these cells [97]. Knocking down Cdh1 in neurons inhibits APC/C-Cdh1 activity and leads PFKFB3 protein to accumulate, thus shifting glucose consumption towards glycolysis at the expense of a reduction in that of the PPP. Importantly, this causes an impairment in neurons to regenerate NADPH(H^+^), hence promoting oxidative stress by promoting the oxidation of antioxidant glutathione [97].

This tight regulation of glycolysis and PPP by APC/C-Cdh1 has important consequences for neuronal survival. Thus, when the activation of the rate of glycolysis is prolonged by inhibiting APC/C-Cdh1 activity, neurons undergo apoptotic death [97]; moreover, neuronal death can be fully reversed by incubation with a plasma membrane-permeable form of glutathione [97]. Thus, glucose is preferentially utilized through the PPP to exert antioxidant-mediated neuroprotection, and APC/C-Cdh1 activity is essential to control this physiological function. Future studies will be needed to understand whether Cdh1 deficiency or the inhibition of APC/C activity exert any role in the mechanism leading to neurodegeneration.

## Abbreviations

APC/C: Anaphase-promoting complex/cyclosome
CNS: Central nervous system
Cdk: Cyclin-dependent kinase
D box: Destruction box
PFKFB3: 6-Phosphofructo-2-kinase/fructose-2,6-bisphosphatase-3
NMDA: *N*-methyl-d-aspartate
PPP: Pentose-phosphate pathway
RING: Really interesting new gene
SCF: Skp1-Cul1-F-box-protein
TGFβ: Transforming growth factor β
TPR: Tetratricopeptide
UPS: Ubiquitin proteasome system
ZBR: Zinc-binding region
